# Development and validation of a machine learning model for in-hospital mortality prediction in children under 5 years with heart failure

**DOI:** 10.3389/fped.2025.1608334

**Published:** 2025-05-26

**Authors:** Huasheng Lv, Fengyu Sun, Teng Yuan, Haoliang Shen, Lazaiyi Baheti, You Chen

**Affiliations:** ^1^ Department of Cardiology, The First Affiliated Hospital of Xinjiang Medical University, Urumqi, China; ^2^ Department of Pediatrics, Xinjiang Medical University, Urumqi, China

**Keywords:** pediatric heart failure, in-hospital mortality, machine learning, risk prediction, interpretability

## Abstract

**Background:**

Heart failure (HF) in children under five years of age carries a high risk of in-hospital mortality, yet existing pediatric risk assessment tools lack specificity for this population. There is a pressing need for reliable, interpretable prediction models tailored to pediatric HF.

**Methods:**

We retrospectively analyzed 630 hospitalized children under five with heart failure from 2013 to 2024. After excluding those with uncorrected congenital heart disease or terminal comorbidities, 67 variables were assessed, and seven key predictors were identified using the Boruta algorithm. Six machine learning models were developed; the Extreme Gradient Boosting (XGB) model was selected and interpreted using SHAP. External validation included 73 additional cases.

**Results:**

The XGB model achieved high predictive performance (AUC: 0.916 training, 0.851 internal validation, 0.846 external validation). The top predictors were NT-proBNP, pH, PCT, LDH, WBC, creatinine, and platelet count. SHAP analysis confirmed the clinical relevance of these variables.

**Conclusion:**

This study presents a reliable, interpretable machine learning model for predicting in-hospital mortality in young children with heart failure. It holds promise for early risk stratification and timely intervention, potentially improving outcomes in this high-risk population.

## 1 Introduction

For Heart failure (HF) in pediatric populations represents a significant global health challenge, contributing substantially to mortality rates among children under five years of age worldwide (1). In young children, the most common underlying etiologies of HF include congenital heart disease and cardiomyopathy (2). Although the overall incidence of pediatric HF is relatively low—estimated between 0.9 and 7.4 cases per 100,000 children annually—the condition carries a markedly high morbidity and mortality burden. Reported in-hospital mortality rates among pediatric HF patients range from 7% to as high as 26%, particularly in younger children or those with complex comorbidities (3, 4). In the United States alone, more than 14,000 pediatric hospitalizations annually are attributed to heart failure, highlighting its substantial clinical impact relative to its low incidence (5). While the absolute burden of HF in children is lower than that observed in adult populations, affected pediatric patients often demonstrate greater severity of illness. Children with HF demonstrate significantly higher resource utilization—including ICU admissions, longer hospital stays, and mechanical circulatory support—than adults with HF. Moreover, mortality rates for children with HF in emergency departments and inpatient settings are frequently higher than those reported for adults, reflecting both the clinical complexity and fragility of the pediatric HF population (6).

These findings highlight the urgent need for improved risk stratification tools tailored to the pediatric HF context, particularly for children under five who are at heightened risk of rapid deterioration. Nonetheless, widely used pediatric risk scores such as PRISM-III and PIM-2 offer limited prognostic insight, as they are not specifically designed for HF (7, 8). This further reinforces the necessity for risk assessment tools specifically tailored to pediatric HF. In recent years, advancements in artificial intelligence have expanded the application of machine learning (ML) in clinical research. ML techniques excel at analyzing complex datasets, enhancing disease diagnosis, prognostication, and treatment outcome prediction. Unlike conventional statistical methods, ML algorithms can decipher intricate nonlinear relationships and uncover previously unrecognized associations, thereby identifying prognostic patterns that may be overlooked by traditional scoring systems (9).

In cardiovascular medicine, ML algorithms have demonstrated success in prognostic prediction for adult populations with HF and other cardiac conditions (10–12). However, research on ML-based risk modeling for pediatric HF remains scarce, with existing models often limited by poor interpretability and generalizability. To address this gap, this study aims to develop and validate an optimized ML-based mortality risk prediction model using a comprehensive dataset to predict in-hospital mortality among children under five years of age with HF. Furthermore, SHapley Additive exPlanations (SHAP) will be employed to improve interpretability by quantifying the contribution of individual clinical features to mortality risk. This dual approach aims to offer a robust, interpretable framework to support clinical decision-making and personalized care in this vulnerable population.

## 2 Methods

### 2.1 Study design and participant

**Figure 1:**
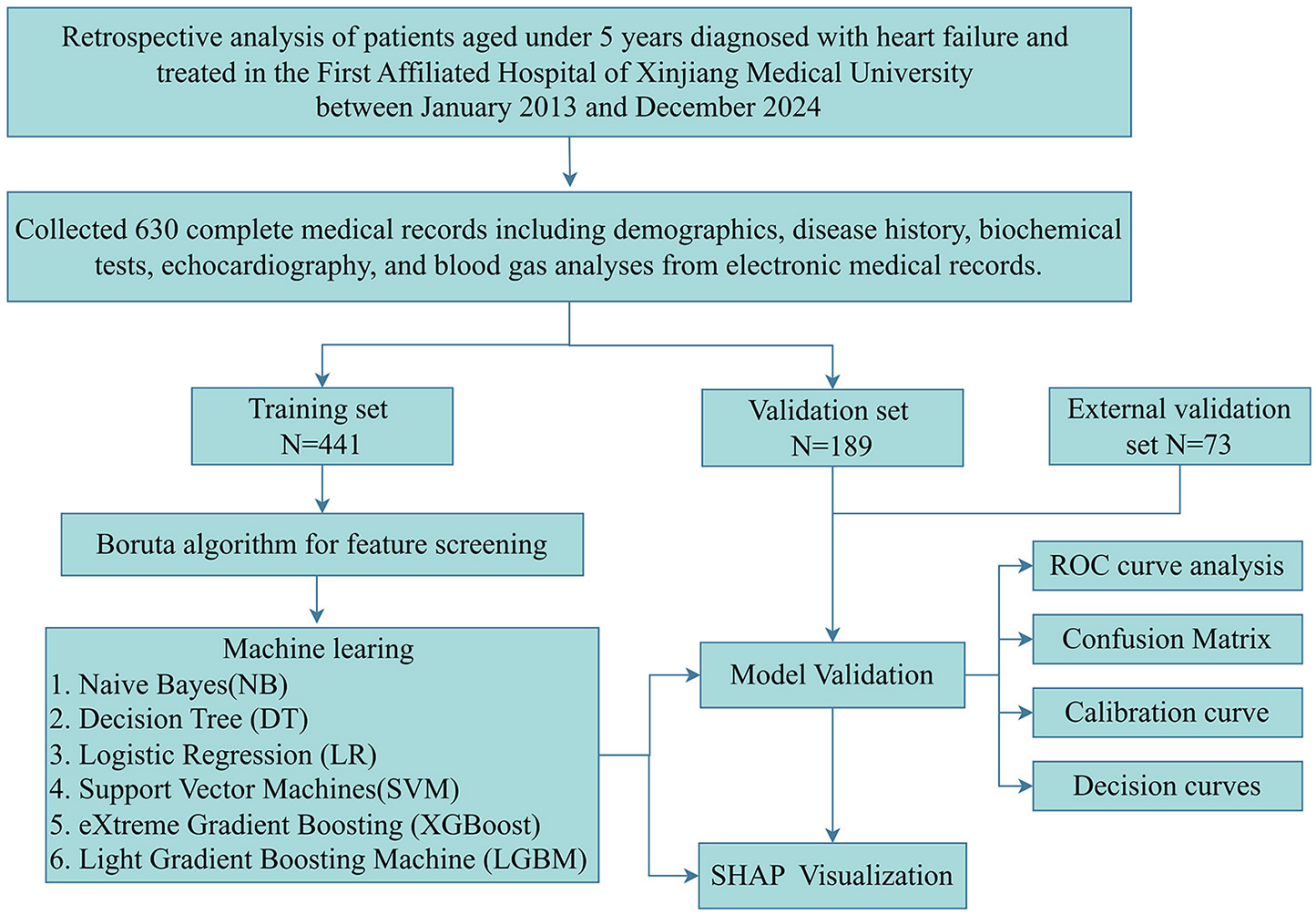
Flowchart of the participants included in the study.

This study enrolled hospitalized children under 5 years with heart failure who were admitted to the First Affiliated Hospital of Xinjiang Medical University between January 2013 and December 2024. The etiologies of heart failure included congenital heart disease, cardiomyopathy, myocarditis, and systemic or inflammatory causes such as severe infection or metabolic derangements. Exclusion criteria comprised: uncorrected major congenital heart disease (physiological single ventricle or unoperated tetralogy of Fallot), non-cardiac terminal illnesses (metastatic malignancies or irreversible genetic disorders), and incomplete medical records. After applying inclusion and exclusion criteria, 630 eligible patients were ultimately enrolled and randomly allocated in a 7:3 ratio to either the training set (*n* = 441) or validation set (*n* = 189). The study protocol received ethical approval from the Institutional Review Board of the First Affiliated Hospital of Xinjiang Medical University (Ethics No.: 20220309-196). All methods were performed in accordance with the relevant guidelines and regulations. Given the retrospective nature of this investigation, the ethics committee waived the requirement for informed consent from pediatric participants and their legal guardians. A comprehensive flowchart detailing participant screening and study procedures is provided in Figure 1.

### 2.2 Data extraction

A total of 67 variables were collected from the electronic medical record system, covering demographic data (sex, ethnicity, age), clinical symptoms (New York Heart Association classification, dyspnea, congenital heart disease, consciousness, edema, cardiac murmur, lung rales, fever), vital signs (heart rate, respiratory rate, blood pressure, body mass index), laboratory tests (hematology, liver and renal function, lipid profile, electrolytes, glucose, lactic dehydrogenase (LDH), N-terminal pro-brain natriuretic peptide (NT-ProBNP), procalcitonin (PCT), coagulation markers, potential of hydrogen (pH), and cardiac ultrasound parameters (stroke volume, cardiac output, atrial and ventricular dimensions, ejection fraction).

### 2.3 Feature screening

In this study, we employed the Boruta algorithm—a robust and widely adopted feature selection method based on random forest—to pre-select features in the training set. The Boruta algorithm identifies key predictive variables by creating shadow features (randomized copies of original features) and evaluating the importance of original features against these shadows using random forest classification. This approach ensures retention of statistically significant variables, while reducing redundancy and overfitting—making it particularly suitable for complex clinical datasets (13, 14).

### 2.4 Model construction and verification

Features selected via Boruta were input into six distinct machine learning models: Naive Bayes (NB), Logistic Regression (LR), Decision Tree (DT), Extreme Gradient Boosting (XGB), Support Vector Machine (SVM), and Light Gradient Boosting Machine (LGBM), to optimize hyperparameters for each algorithm.

Model performance was evaluated using confusion matrix metrics, including accuracy, the area under the curve (AUC) of the receiver operating characteristic (ROC) curve, recall, specificity, and the Brier score. The Brier score quantifies the magnitude of deviation between predicted and actual outcomes, with lower values indicating superior predictive performance (15). ROC curve analysis and AUC comparisons were conducted to identify the highest-performing model, complemented by decision curve analysis and calibration curves to assess clinical utility.

Feature importance ranking was performed to quantify the contribution of individual variables to model outcomes. Shapley values from cooperative game theory were applied to determine each input variable's influence on model predictions (16, 17). To address the interpretability challenges inherent in machine learning models, SHAP was applied to the optimal model to quantify the contribution of each feature, thereby enhancing clinical transparency and understanding. Global SHAP values were visualized as bar plots to illustrate the average impact of each feature. SHAP was utilized to elucidate predictions generated by the optimal model.

### 2.5 External validation

For external validation, 73 cases (including 11 deaths) of children under 5 years with heart failure treated at Urumqi Youai Hospital were enrolled in the external validation cohort. The external validation cohort was derived from different hospitals within the same geographical region, demonstrating clinical characteristics comparable to those of the training set. We applied the data from the external validation set to the model constructed from the training set, subsequently evaluating the model's performance, goodness-of-fit, and clinical utility through the generation of ROC curves, calibration curves, and decision curve analysis curves.

### 2.6 Statistical analysis

Data processing and statistical analyses were performed using R (version 4.4.2) and Python (version 3.11.7). Continuous variables with normal distributions are presented as mean ± standard deviation, while skewed data are reported as median and interquartile range. Student's t-test was applied for normally distributed continuous variables, and the Mann–Whitney U-test for non-parametric comparisons. Categorical variables are expressed as percentages or frequencies, with group differences assessed via chi-square tests. Statistical significance levels were established at *P* < 0.05.

## 3 Results

### 3.1 Patient characteristics

Based on inclusion and exclusion criteria, 630 eligible patients were enrolled and divided into training and validation sets at a 7:3 ratio (Supplementary Table S1). Among the 630 children under 5 years with heart failure included in the analysis, 91 experienced in-hospital mortality, yielding a mortality rate of 14%. Significant differences were observed between survivors and non-survivors across multiple parameters (Table 1). The Spearman correlation analysis method was used to evaluate inter-indicator correlations within the models (Supplementary Figure S1).

**Table 1:** Baseline characteristics of children under 5 years with heart failure.

Variables	Survival group (*n* = 539)	Death group (*n* = 91)	*P*
Sex, %			0.239
Female	249 (46.2)	36 (39.6)	
Male	290 (53.8)	55 (60.4)	
Ethnic group, %			0.053
Han Chinese	186 (34.5)	41 (45.1)	
Ethnic minorities	353 (65.5)	50 (54.9)	
Age (years)	0.7 (0.3, 1.7)	0.6 (0.3, 2.1)	0.897
NYHA, %			<0.001
1	69 (12.8)	18 (19.8)	
2	258 (47.9)	18 (19.8)	
3	41 (7.6)	21 (23.1)	
4	171 (31.7)	34 (37.4)	
Dyspnea, %			0.506
No	403 (74.8)	71 (78)	
Yes	136 (25.2)	20 (22)	
CHD, %			0.393
No	475 (88.1)	83 (91.2)	
Yes	64 (11.9)	8 (8.8)	
Consciousness, %			0.105
No	467 (86.6)	73 (80.2)	
Yes	72 (13.4)	18 (19.8)	
LE-Edema, %			0.166
No	502 (93.1)	81 (89)	
Yes	37 (6.9)	10 (11)	
Card-Murmur, %			0.698
No	344 (63.8)	60 (65.9)	
Yes	195 (36.2)	31 (34.1)	
Lung-Moist, %			0.638
No	264 (49)	47 (51.6)	
Yes	275 (51)	44 (48.4)	
HR (bpm)	145.4 ± 28.1	143.1 ± 33.8	0.480
Fever, %			0.359
No	364 (67.5)	57 (62.6)	
Yes	175 (32.5)	34 (37.4)	
RR (breaths/min)	40.1 ± 14.2	37.8 ± 14.7	0.145
DBP (mmHg)	55.8 ± 12.5	54.7 ± 16.6	0.470
SBP (mmHg)	91.5 ± 15.1	92.3 ± 18.8	0.644
BMI (kg/m^2^)	15.4 ± 2.7	15.9 ± 2.5	0.094
WBC (×10⁹/L)	11.4 (8.0, 15.1)	12.2 (7.9, 18.5)	0.085
RBC (×10^12^/L)	4.1 ± 0.9	3.9 ± 1.0	0.035
Lymph (×10⁹/L)	4.5 (2.9, 6.7)	4.9 (2.6, 6.8)	0.938
Mono (×10⁹/L)	0.8 (0.5, 1.2)	0.8 (0.5, 1.5)	0.837
Neut (×10⁹/L)	4.3 (2.4, 7.3)	5.5 (2.5, 8.8)	0.140
Hb (g/L)	103.1 ± 22.4	97.1 ± 24.7	0.021
PLT (×10⁹/L)	348.6 ± 167.1	277.1 ± 161.1	<0.001
ALT (U/L)	26.0 (18.0, 45.7)	29.0 (15.5, 51.2)	0.846
AST (U/L)	47.3 (34.8,72.1)	47.1 (36.2,105.3)	0.208
GGT (U/L)	25.3 (14.0, 48.7)	28.2 (12.4, 65.7)	0.757
DBIL (μmol/L)	1.6 (0.3, 3.3)	3.2 (1.0, 5.3)	<0.001
IBIL (μmol/L)	6.8 (4.2, 10.9)	6.8 (4.3, 12.7)	0.554
ALB (g/L)	36.6 ± 7.0	34.3 ± 7.6	0.004
GLO (g/L)	24.6 ± 6.4	23.7 ± 7.2	0.237
Crea (μmol/L)	25.1 (19.4, 33.0)	28.0 (20.6, 40.7)	0.017
UA (μmol/L)	273.5 ± 152.2	332.8 ± 182.7	<0.001
TC (mmol/L)	3.1 ± 1.3	2.8 ± 1.3	0.047
TG (mmol/L)	1.3 (0.9, 1.7)	1.1 (0.8, 1.6)	0.091
HDL-C (mmol/L)	0.8 ± 0.4	0.8 ± 0.4	0.157
LDL-C (mmol/L)	1.9 ± 1.0	1.7 ± 0.9	0.029
K^+^ (mmol/L)	4.1 ± 0.7	4.3 ± 1.2	0.022
Na^+^ (mmol/L)	136.4 ± 5.9	135.8 ± 6.3	0.440
Cl^−^ (mmol/L)	102.6 ± 7.0	102.9 ± 7.4	0.697
Ca (mmol/L)	2.3 ± 0.3	2.2 ± 0.3	0.009
*P* (mmol/L)	1.6 ± 0.5	1.7 ± 0.8	0.127
Mg^2+^ (mmol/L)	0.9 ± 0.2	1.0 ± 0.2	0.002
Glu (mmol/L)	5.6 ± 2.5	5.6 ± 3.1	0.804
LDH (U/L)	349.5 (270.3, 527.0)	397.6 (283.6, 766.2)	0.006
ALP (U/L)	174.8 (123.3, 247.1)	160.0 (109.9, 243.8)	0.408
ChE (U/L)	5,563.1 ± 2,129.2	4,898.2 ± 2,224.7	0.006
Cys-C (μmol/L)	1.2 ± 0.5	1.2 ± 0.7	0.153
TT (s)	24.7 ± 4.5	27.5 ± 11.3	<0.001
PT (s)	14.2 ± 8.7	16.7 ± 8.6	0.011
D-Dimer (μg/L)	617.0 (285.5, 1,657.5)	818.0 (465.5, 2,535.5)	0.005
pH	7.4 ± 0.2	7.3 ± 0.2	<0.001
GSP (mmol/L)	2.1 ± 0.6	2.1 ± 0.5	0.819
PCT (ng/ml)	0.2 (0.1, 1.4)	0.5 (0.1, 3.6)	<0.001
NT-ProBNP (pg/ml)	2,646.0 (1,403.5, 3,892.0)	6,661.0 (4,129.0, 8,467.0)	<0.001
CK (U/L)	85.0 (46.5, 181.0)	105.3 (50.1, 254.1)	0.084
Ccr (ml/min)	50.7 (32.5, 73.2)	43.0 (25.3, 81.2)	0.385
GFR (ml/min/1.73m^2^)	750.4 (459.6, 1,089.5)	647.5 (393.7, 937.4)	0.050
SV (ml)	16.3 ± 10.6	16.7 ± 10.2	0.755
CO (L/min)	2.0 ± 1.2	2.0 ± 1.1	0.990
LAD (mm)	17.5 ± 5.4	17.4 ± 5.6	0.889
LVESD (mm)	17.7 ± 7.2	16.8 ± 6.1	0.298
LVEDD (mm)	25.9 ± 8.2	25.5 ± 7.4	0.618
LVEF (%)	63.4 ± 12.5	65.8 ± 10.2	0.079
IVS Thickness (mm)	4.3 ± 1.3	4.5 ± 1.4	0.113
LVPW Thickness (mm)	4.2 ± 1.2	4.4 ± 1.3	0.245
RAD (mm)	19.2 ± 5.8	19.3 ± 6.6	0.827
RV Internal Dim (mm)	11.4 ± 3.4	11.1 ± 3.1	0.565

Data are mean ± SD or *N* (%). NYHA, New York Heart Association; CHD, congenital heart disease; HR, heart rate; RR, respiratory rate; DBP, diastolic blood pressure; SBP, systolic blood pressure; BMI, body mass index; WBC, white blood cell count; RBC, red blood cell count; Lymph, lymphocyte count; Mono, monocyte count; Neut, neutrophil count; Hb, hemoglobin; PLT, platelet count; ALT, alanine transaminase; AST, aspartate transaminase; GGT, gamma-glutamyl transferase; DBIL, direct bilirubin; IBIL, indirect bilirubin; ALB, albumin; GLO, globulin; Crea, creatinine; UA, uric acid; TC, total cholesterol; TG, triglyceride; HDL-C, high-density lipoprotein cholesterol; LDL-C, low-density lipoprotein cholesterol; K^+^, potassium; Na^+^, sodium; Cl^−^, chloride; Ca, calcium; P, phosphorus; Mg^2+^, magnesium; Glu, glucose; LDH, lactic dehydrogenase; ALP, alkaline phosphatase; ChE, cholinesterase; Cys-C, cystatin C; TT, thrombin time; PT, prothrombin time; pH, potential of hydrogen; GSP, glycated serum protein; PCT, procalcitonin; NT-ProBNP, N-terminal pro-brain natriuretic peptide; CK, creatine kinase; Ccr, creatinine clearance; GFR, glomerular filtration rate; SV, stroke volume; CO, cardiac output; LAD, left atrial diameter; LVESD, left ventricular end-systolic diameter; LVEDD, left ventricular end-diastolic diameter; LVEF, left ventricular ejection fraction; IVS Thickness, interventricular septum thickness; LVPW Thickness, left ventricular posterior wall thickness; RAD, right atrial diameter; RV Internal Dim, right ventricular internal dimension.

### 3.2 Feature selection

**Figure 2:**
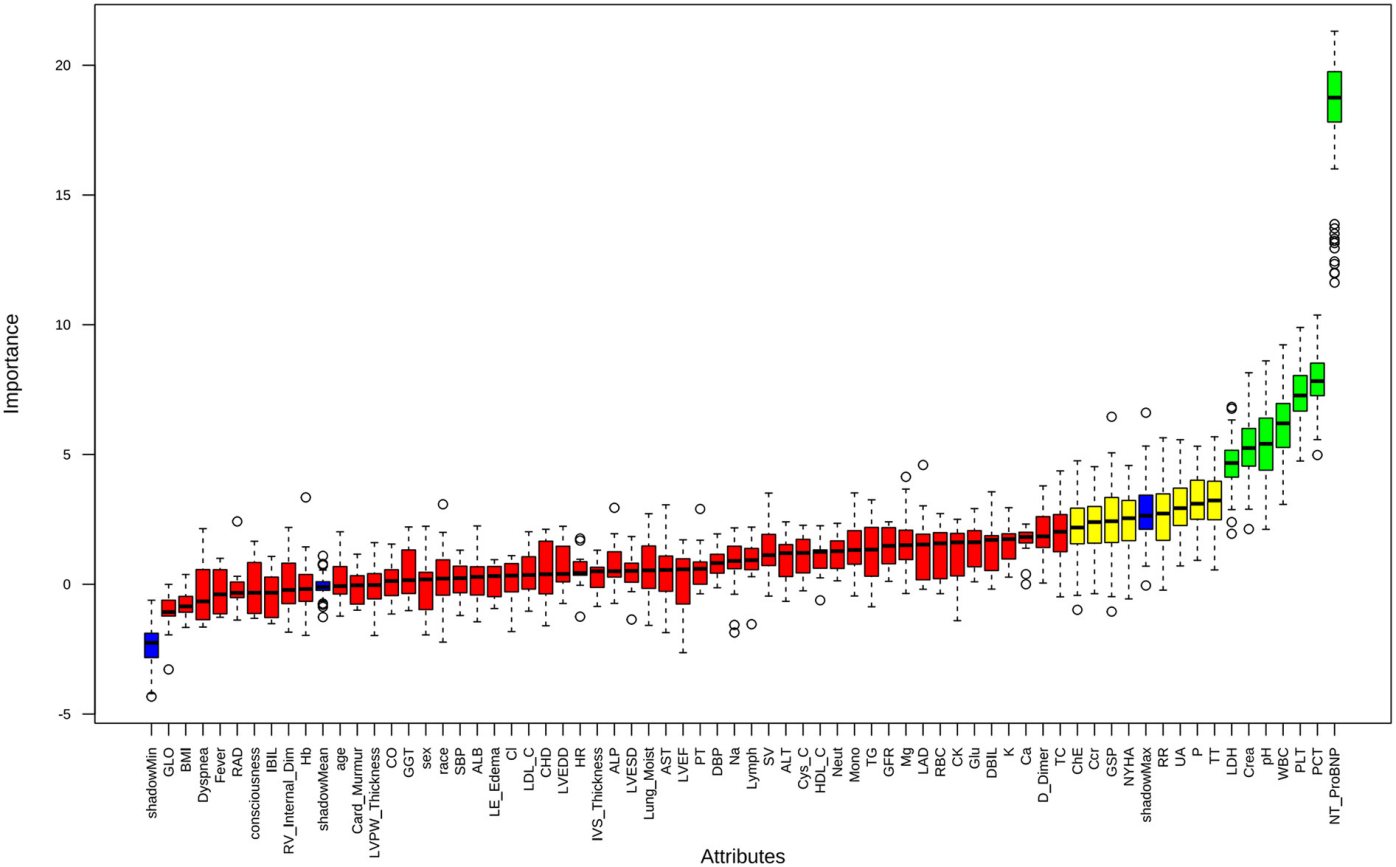
Feature selection based on Boruta algorithm. Green represents acceptable variables.

**Figure 3:**
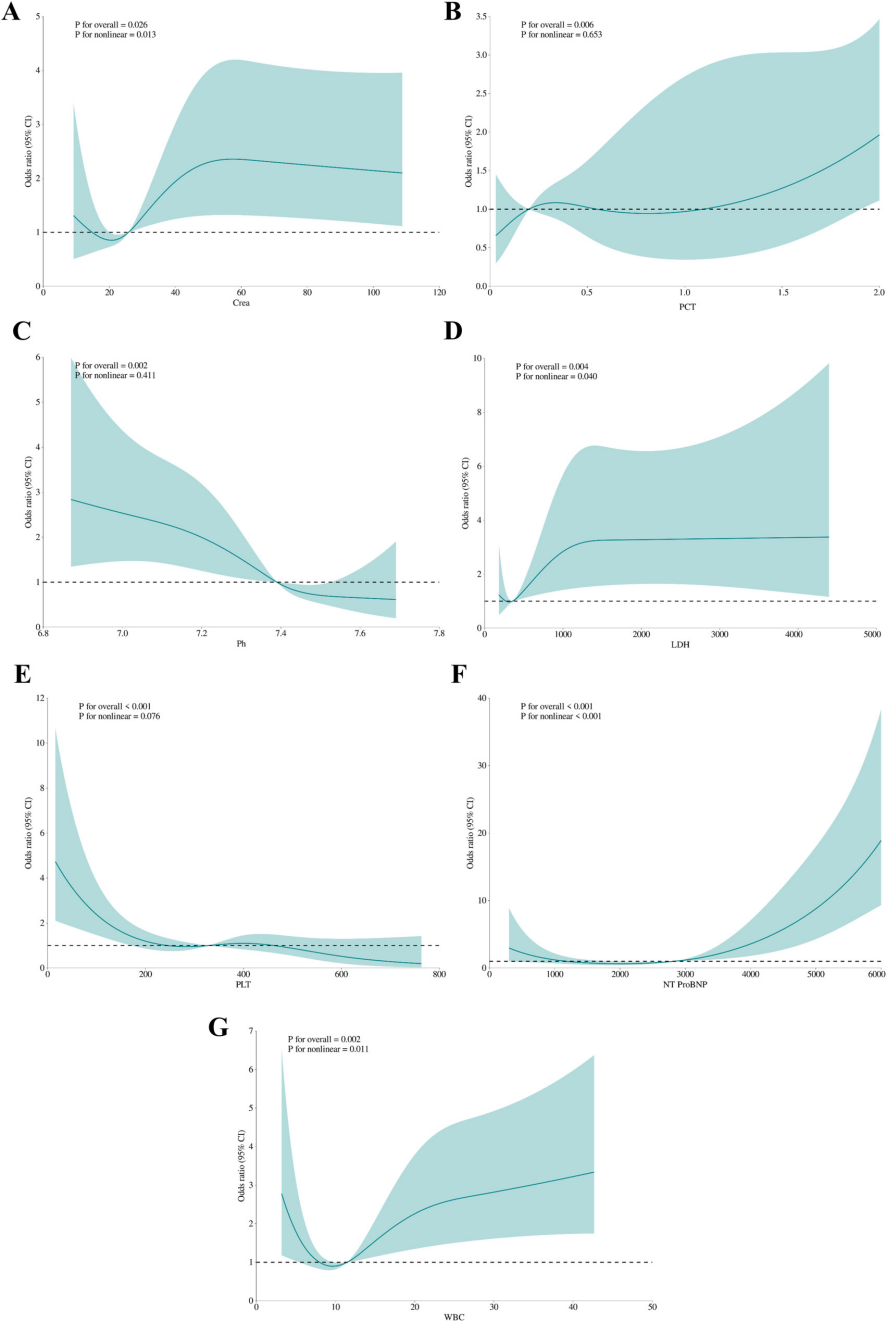
The association between variables and hospital mortality. Creatinine **(A)**, PCT **(B)**, pH **(C)**, LDH **(D)**, PLT **(E)**, NT-proBNP **(F)**, WBC **(G)**: the restricted cubic splines with four knots. The horizontal dashed line represents the reference OR of 1.0.

Feature selection using the Boruta algorithm identified seven optimal predictors from 67 candidate features: NT-ProBNP, platelet count (PLT), pH, LDH, PCT, creatinine, and white blood cell count (WBC) (Figure 2). These variables demonstrated stable predictive value across multiple model iterations (Supplementary Figure S2). Restricted cubic spline analysis revealed the following associations (Figure 3): creatinine exhibited a positive correlation with mortality (*P* = 0.026), with a steep initial rise followed by stabilization; PCT showed a U-shaped relationship with mortality (*P* = 0.006), though its nonlinear association was not significant (*P* = 0.653); LDH, NT-ProBNP, and WBC were positively correlated with mortality, while pH inversely correlated with mortality (*P* = 0.002), displaying a gradual decline from higher values. PLT demonstrated a decreasing association with mortality within the 0–400 range before stabilizing, with no significant nonlinear relationship (*P* = 0.076), suggesting differential effects depending on its levels.

### 3.3 Performance of 6 machine learning prediction models

**Figure 4:**
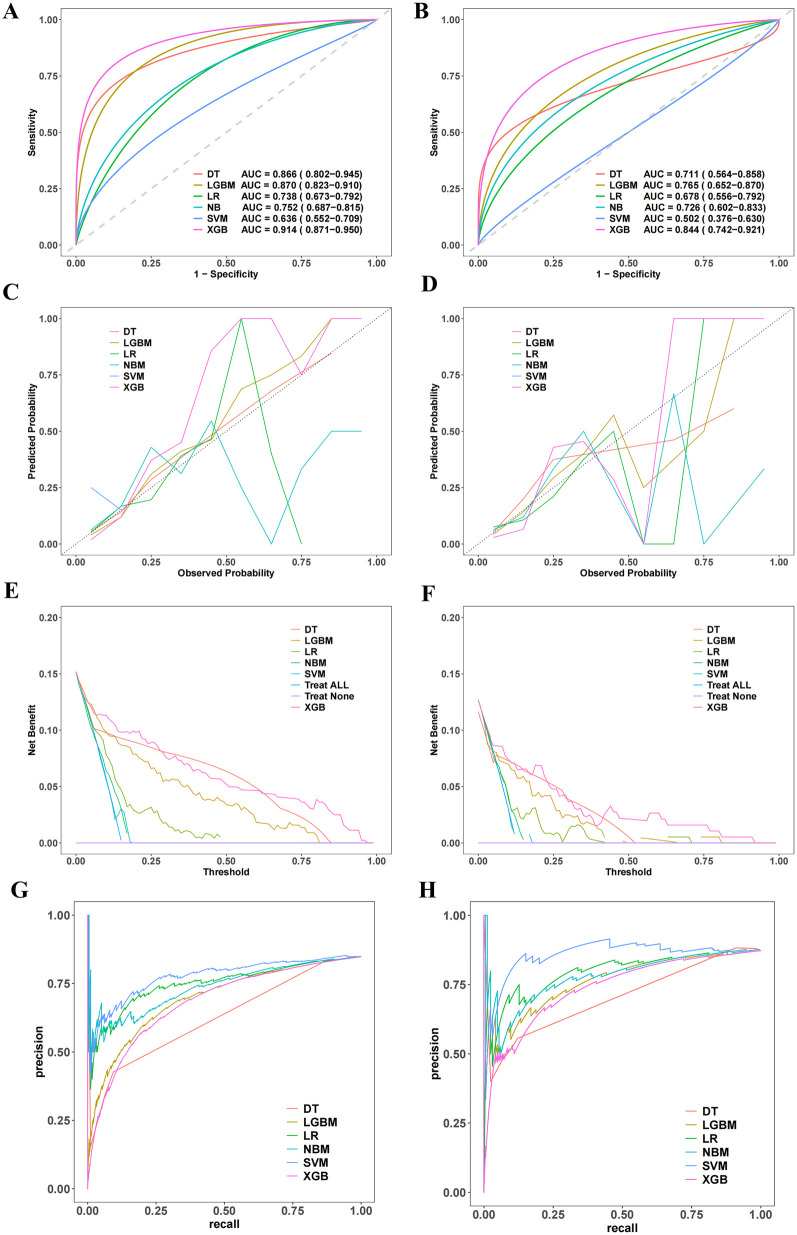
Establishment and validation of the machine learning prediction model. **(A,B)** Present the ROC curves. **(C,D)** Show the calibration curves. **(E,F)** Display the decision curve analysis. **(G,H)** Illustrate the recall-precision curves.

**Figure 5:**
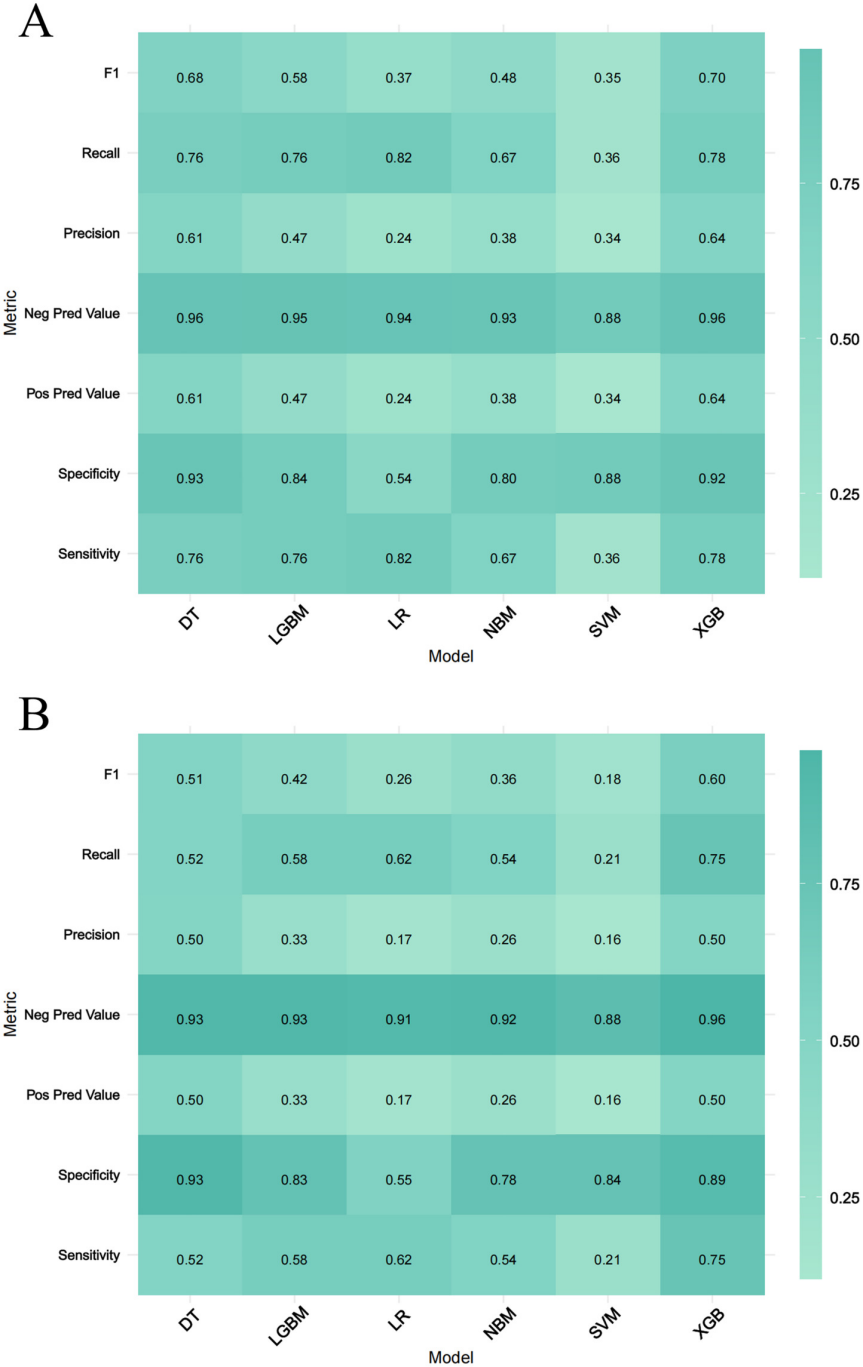
Performance metrics comparison across Six machine learning models. **(A,B)** Represent the training set and the validation set, respectively. The evaluated metrics include F1 score, recall, precision, negative predictive value (Neg Pred value), positive predictive value (Pos Pred value), specificity, and sensitivity.

Six machine learning models—XGB, LGBM, SVM, DT, LR, and NBM—were developed to predict in-hospital mortality. After hyperparameter tuning, models were trained on the training set and evaluated on the validation set. Among these, the XGB model achieved the highest performance, with training and validation AUCs of 0.916 and 0.851, respectively (Figures 4A,B). Calibration curves confirmed strong alignment between predicted probabilities and observed outcomes for XGB (Figures 4C,D), while decision curve analysis demonstrated superior clinical net benefit across most threshold probabilities (Figures 4E,F). Precision-recall curves further validated the model's robustness (Figures 4G,H). In the training set, XGB exhibited a Brier score of 0.072, sensitivity of 0.776, specificity of 0.922, and F1-score of 0.703; corresponding validation metrics were 0.080, 0.750, 0.891, and 0.600, respectively (Figure 5 and Table 2). The lowest Brier score indicated high accuracy and generalizability for mortality prediction. A violin plot comparing feature importance across models highlighted XGB and DT as the strongest predictors of mortality, with XGB showing the highest reliability (peak value: 0.4; confidence interval: up to 1.0) (Supplementary Figure S3). XGB was ultimately selected as the optimal model.Further technical details regarding the XGB model's configuration, performance metrics, and analytical considerations are provided in Supplementary Material 1.

**Table 2:** Predictive performances of six machine learning models in training and validation sets for in-hospital mortality prediction.

Model	AUC (95% CI)	Brier score	Sensitivity	Specificity	Precision	Recall	F1	Pos Pred value	Neg Pred value
Training set									
LR	0.732 (0.670, 0.794)	0.117	0.821	0.54	0.242	0.821	0.374	0.242	0.944
DT	0.830 (0.770, 0.889)	0.075	0.759	0.933	0.612	0.759	0.678	0.612	0.965
XGB	0.916 (0.878, 0.954)	0.072	0.776	0.922	0.642	0.776	0.703	0.642	0.958
SVM	0.637 (0.560, 0.715)	0.128	0.358	0.877	0.343	0.358	0.35	0.343	0.884
LGBM	0.871 (0.826, 0.916)	0.088	0.761	0.845	0.468	0.761	0.58	0.468	0.952
NBM	0.769 (0.706, 0.832)	0.124	0.672	0.799	0.375	0.672	0.481	0.375	0.931
Validation set									
LR	0.683 (0.560, 0.806)	0.104	0.625	0.552	0.169	0.625	0.265	0.169	0.91
DT	0.724 (0.577, 0.870)	0.092	0.522	0.928	0.5	0.522	0.511	0.5	0.933
XGB	0.851 (0.760, 0.942)	0.080	0.75	0.891	0.5	0.75	0.6	0.5	0.961
SVM	0.516 (0.382, 0.651)	0.111	0.208	0.836	0.156	0.208	0.179	0.156	0.879
LGBM	0.778 (0.665, 0.890)	0.093	0.583	0.83	0.333	0.583	0.424	0.333	0.932
NBM	0.736 (0.618, 0.854)	0.108	0.542	0.782	0.265	0.542	0.356	0.265	0.921
External validation set									
XGB	0.846 (0.538–1.000)	0.069	0.500	1.000	1.000	0.500	0.667	1.000	0.929

### 3.4 Model interpretability

**Figure 6:**
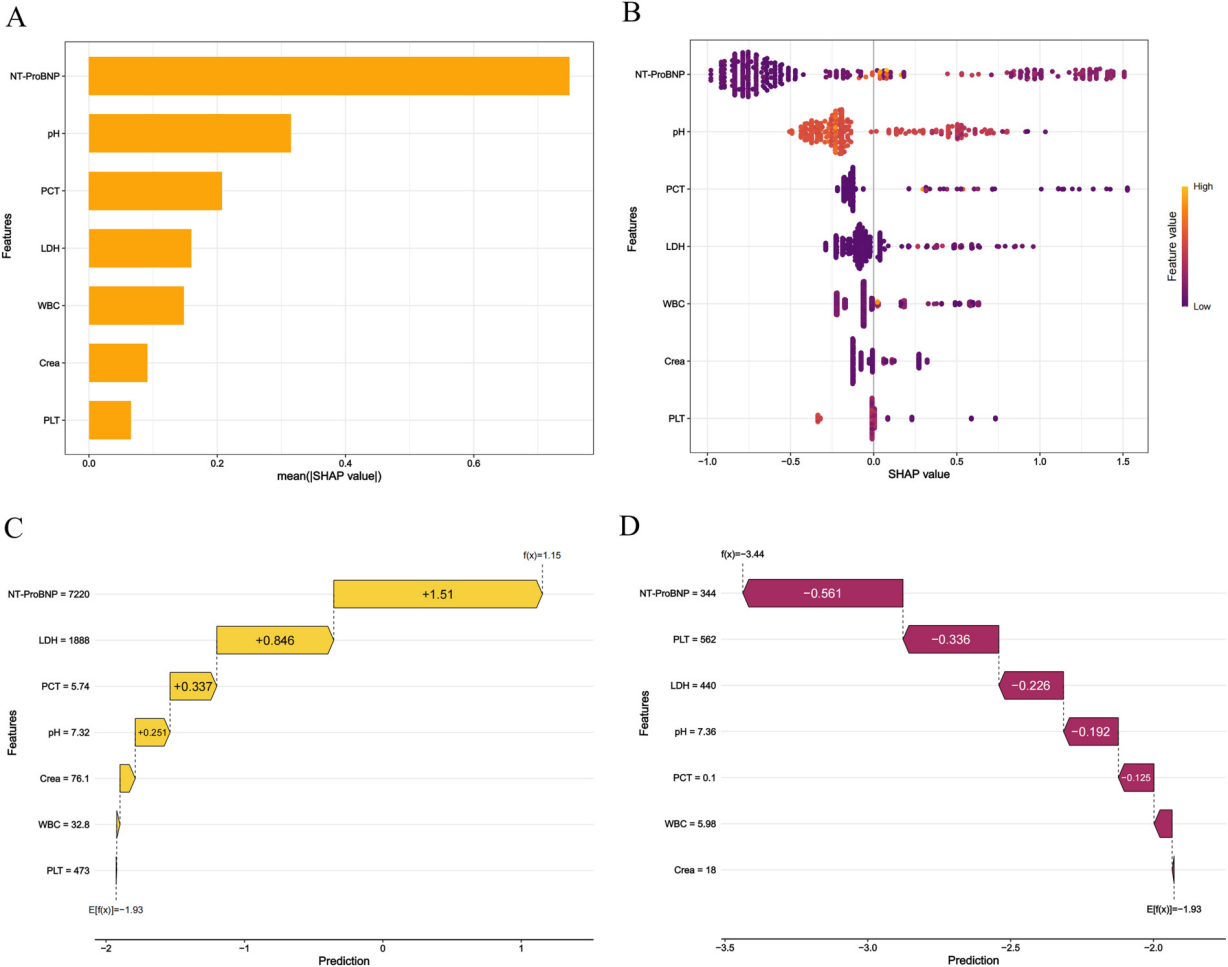
The shapley additive exPlanations values of the best prediction model, XGB. **(A)** Average impact of features on model predictions. **(B)** Detailed impact analysis of each feature SHAP interpretation of the XGB model. Every dot in a row symbolizes a patient, and its color denotes the feature value—yellow denotes a value that is greater and purple denotes a value that is lower. The more dispersed the points of the graph represent the greater the impact of the variables on the model. **(C,D)** Personalized predictions for a patient. The risk and protective variables are symbolized by the yellow and plum bars. Higher functional significance is indicated by longer bars.

SHAP analysis was applied to visualize feature contributions in the XGB model. The mean absolute SHAP values (Figure 6A) ranked NT-ProBNP as the most influential predictor, followed by pH, PCT, LDH, WBC, creatinine, and PLT. Directional impacts of features on individual predictions are detailed in Figure 6B, where yellow bars indicate features increasing mortality risk and purple bars denote protective effects. For example, in a deceased patient (Figure 6C), elevated NT-ProBNP (7,220; SHAP +1.51), LDH (1,888; +0.846), PCT (5.74; +0.337), and pH (7.32; +0.251) collectively shifted the prediction toward mortality {final output: *f* (*x*) = 1.15; baseline: *E* [*f* (*x*)] = −1.93}. Conversely, in a survivor (Figure 6D), lower NT-ProBNP (1,900; SHAP −0.755), higher PLT (535; −0.336), and other favorable values reduced mortality risk [final output: *f* (*x*) = −3.65].

### 3.5 External validation

**Figure 7:**
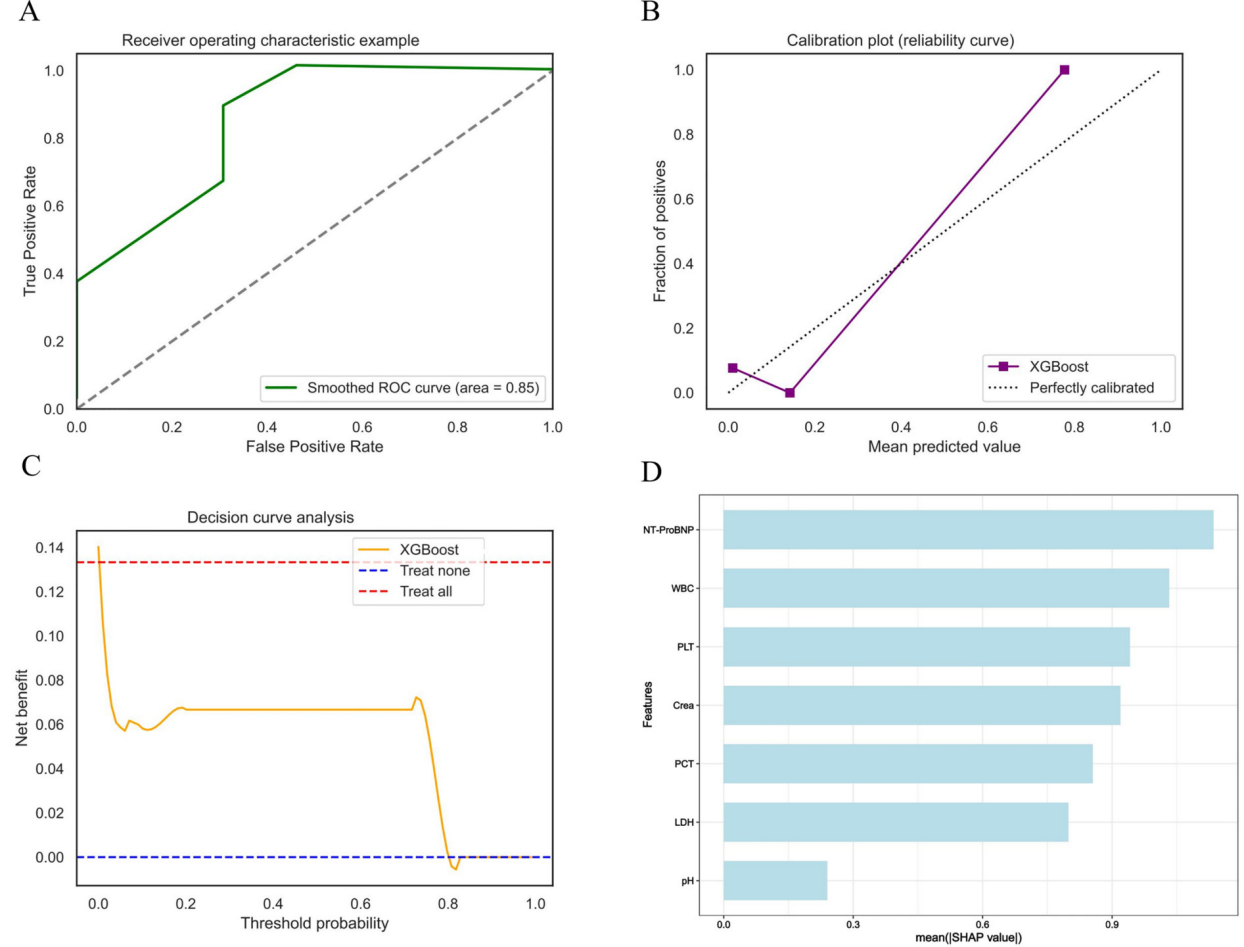
External validation shows excellent performance in models. **(A)** XGB validation set ROC curve. **(B)** XGB validation set calibration curve. **(C)** XGB validation set decision curve analysis curve. **(D)** Average impact of features on model predictions.

To verify the model's generalization and practical value, an external hospital database was used (Supplementary Table S2). The model showed superior predictive performance: the ROC curve (Figure 7A) had an AUC of 0.85, demonstrating good discrimination for death risk; the calibration curve (Figure 7B) showed the XGB model's predicted mortality probability aligned with actual outcomes. The decision curve analysis curve (Figure 7C) indicated the model provided higher net clinical benefit than “no intervention” and “universal intervention” strategies in predicting mortality. Figure 7D's feature analysis via mean SHAP values visualized contributions of key predictors, guiding clinical decisions on death risk assessment. The model demonstrated favorable performance across key metrics including brier score, sensitivity, specificity, and F1 score (Table 2).

## 4 Discussion

In this study, we developed an XGB machine learning model to predict in-hospital mortality among children under five years old with HF, and demonstrated robust performance across both internal and external validation cohorts. The model achieved an AUC of 0.916 in the training set and 0.851 in the internal validation set, significantly outperforming other commonly used models. Notably, performance remained stable in an external validation cohort (AUC = 0.846), indicating strong generalizability and the ability to effectively discriminate between survivors and non-survivors in this high-risk population. This level of accuracy is comparable to, or exceeds, that reported in previous pediatric mortality prediction studies. For instance, Du et al. reported an XGB-based model that achieved a sensitivity of 78.5% and specificity of 82.4% for predicting postoperative mortality in children with congenital heart disease. Our model demonstrated similar performance, suggesting that it is capable of identifying the majority of fatal cases while minimizing false positives among survivors (18). Such performance represents a notable improvement over traditional logistic regression-based tools (7) and highlights the added value of machine learning in integrating and analyzing complex pediatric datasets.

Beyond overall accuracy, an important finding lies in the model's identification of top predictive features: notably NT-proBNP, blood pH, PCT, LDH, WBC, creatinine, and PLT. These variables reflect well-established pathophysiological mechanisms associated with critical illness and offer insight into risk stratification in young HF patients. NT-proBNP emerged as the most influential predictor—a result aligned with extensive evidence of its prognostic utility in both adult and pediatric heart failure (19). The American Heart Association has emphasized NT-proBNP as a key biomarker for assessing severity and prognosis in pediatric HF (20). In a study by Chowdhury et al., an NT-proBNP level ≥520.2 pg/ml predicted moderate-to-severe HF (≥ class II) with 83% sensitivity and 91% specificity. Median NT-proBNP in non-survivors (11,681.01 pg/ml) was significantly higher than in survivors (839.4 pg/ml, *p* < 0.001) (21). A recent meta-analysis further confirmed the association between elevated NT-proBNP and increased mortality risk, reporting a hazard ratio of 1.65 (95% CI: 1.55–1.76) (22). The strong contribution of NT-proBNP in our model reinforces its clinical relevance. A child presenting with markedly elevated levels should be regarded as high-risk and considered for early intensive management or ICU monitoring. Likewise, blood pH was identified as a top feature, indicating the prognostic relevance of metabolic acidosis. A recent review highlighted compelling evidence linking critically low arterial pH (mean 6.15) to sudden infant death, underscoring its role in reflecting severe physiological instability and end-organ failure (23).

Inflammatory and tissue injury markers also featured prominently. PCT, a well-known biomarker of bacterial infection and sepsis, is frequently elevated during systemic inflammatory responses (24). Its inclusion in our model suggests that infectious complications or systemic inflammation may be common precipitants of decompensation in pediatric HF. Elevated PCT levels on admission may help identify patients in early sepsis or with infections exacerbating cardiac dysfunction. Prior studies have shown that high PCT is associated with increased risk of organ dysfunction and mortality in critically ill pediatric populations (25, 26). Similarly, LDH—a nonspecific enzyme released during tissue breakdown or hypoxia—was identified as a strong predictor. LDH often increases in conditions involving multi-organ injury, hemolysis, or hepatic congestion, all of which are common in advanced heart failure (27, 28). In a cohort of 4,343 critically ill children, Wang et al. found that LDH had the highest predictive accuracy for in-hospital mortality (AUC = 0.729) and remained independently associated with death after adjusting for age and organ dysfunction (OR = 2.45, 95% CI: 1.84–3.24) (29). Furthermore, LDH was significantly associated with 30-day mortality and ICU length of stay, surpassing traditional cardiac biomarkers in predictive performance. The combination of low pH and elevated LDH effectively captured the clinical profile of systemic shock and widespread cellular injury. This represents an especially ominous pattern in children with underlying HF.

Creatinine elevation reflects impaired renal function, which in HF patients may result from low cardiac output or venous congestion (30). A landmark meta-analysis of 16 studies involving over 80,000 HF patients reported a 15% increased risk of mortality per 0.5 mg/dl increase in creatinine, with more than a twofold mortality risk in patients with moderate-to-severe renal dysfunction (creatinine ≥1.5 mg/dl) (31). Our findings confirm that elevated creatinine in children is associated with increased mortality risk, likely reflecting severe hemodynamic compromise, renal hypoperfusion, or concurrent nephrotoxic injury.

Elevated WBC count is commonly linked to systemic inflammation or infection, which are frequent precipitants of decompensation in pediatric heart failure. In addition to WBC, platelet count has shown independent prognostic significance. A large retrospective analysis of the MIMIC-IV database revealed that thrombocytopenia was strongly associated with 28-day mortality in sepsis patients, with a nearly twofold increase in risk for those with platelet counts below 50 × 10^9^/L (32). Notably, WBC and platelet indices can be jointly interpreted as markers of the host hematologic response to critical illness. The platelet-to-white cell ratio has been shown to outperform other complete blood count–derived indices in predicting mortality in several acute inflammatory diseases, including acute heart failure. Lower platelet-to-white cell ratio, reflecting elevated WBC and/or thrombocytopenia, was significantly associated with short-term mortality and in some cohorts exceeded age in prognostic relevance (33). Collectively, these findings illustrate that our model not only identifies clinically relevant biomarkers associated with pediatric HF mortality but also mirrors established physiological mechanisms, reinforcing its potential as a clinically meaningful tool.

A key strength of our study was the use of SHAP analysis to enhance transparency and bridge the gap between algorithmic predictions and clinical reasoning. A common criticism of machine learning in clinical contexts is the “black box” nature of many algorithms, which can limit clinician trust and uptake (34). By applying SHAP, we were able to deconstruct each prediction into individual feature contributions, thereby improving transparency in decision-making (35). For example, elevated NT-proBNP and low pH may emerge as dominant contributors to a high-risk classification, whereas normal WBC count may offset risk. This level of interpretability ensures that the model's predictions are aligned with established clinical reasoning, increasing its potential for integration into real-world practice.

When embedded within hospital systems, interpretable ML models like ours could actively support real-time decision-making by alerting clinicians to high-risk pediatric HF patients and guiding timely intervention. In resource-limited environments, ML-powered triage systems could help allocate scarce critical care resources—such as ICU beds or surgical capacity—to those pediatric patients most likely to benefit (36, 37). At a broader level, such tools may support global health equity by addressing preventable HF-related mortality among children under five. Given that our model relies solely on routinely collected laboratory and clinical variables, it can be integrated into electronic health record (EHR) systems to generate automated risk scores at the point of admission. Such implementation could allow frontline providers to prioritize high-risk patients early and streamline escalation-of-care decisions. Our model thus functions not only as an early warning system, but also as a strategic tool for informing both clinical practice and policy development aimed at achieving Sustainable Development Goal 3.2 (38).

Although the model performed well, certain methodological limitations remain. Boruta may underrepresent weak predictors in imbalanced data, and early stopping in XGB, while helpful, does not fully eliminate overfitting risk given the low event rate.

## 5 Limitations

Despite the promising results, our study has several important limitations. First, it was retrospective in nature, relying on observational medical record data. This introduces potential for missing data, documentation errors, and residual confounding, and limits causal inference. Second, although we performed external validation, the size of the external cohort was relatively small. Larger and more heterogeneous validation cohorts—across multiple institutions and geographic regions—are needed to confirm generalizability. Further multi-center validation studies are currently being planned. Third, the presence of unmeasured confounders cannot be excluded. Variables such as nutritional status, pre-admission medication history, and timing of hospital presentation may influence disease severity, treatment decisions, and outcomes, potentially affecting model performance. Lastly, only basic echocardiographic parameters (e.g., ejection fraction, chamber size) were consistently available, while advanced imaging metrics (e.g., strain imaging, tissue Doppler, cardiac MRI) were largely missing and thus excluded, which may limit the model's physiological depth.

## 6 Conclusion

This study developed and externally validated an XGB-based model to predict in-hospital mortality in children under five with heart failure, achieving high accuracy and generalizability. By identifying clinically meaningful predictors and incorporating SHAP analysis, the model offers both predictive performance and interpretability. These findings support its potential as a clinical decision-support tool for early risk stratification, guiding interventions and resource allocation. Further prospective studies are warranted to confirm its utility across diverse healthcare settings.

## Data Availability

The raw data supporting the conclusions of this article will be made available by the authors, without undue reservation.
