# Supplementary material for: Development and validation of a machine learning model for in-hospital mortality prediction in children under 5 years with heart failure
Source: Front Pediatr. 2025 May 26;13:1608334. doi: 10.3389/fped.2025.1608334 (PMC12146293; doi:10.3389/fped.2025.1608334)
Supplement: Supplementary file 1 [file Datasheet1.docx]

Supplementary Material

**Supplementary Material 1.: Feature Selection and Model Training Parameters**.

# 1. Feature Selection (Boruta)

# The Boruta algorithm was used to identify all-relevant predictors. The algorithm was iterated 100 times to ensure stability and robustness in feature selection.

# 2. XGB Model Configuration

# The final model was optimized with the following hyperparameters:

# eta = 0.3

# max_depth = 2

# subsample = 0.7

# colsample_bytree = 0.4

# objective = binary:logistic

# Early stopping was employed with a maximum of 200 rounds. The best iteration was selected at round 17 based on test log-loss (minimum = 0.2756).

# 3. Model Performance Metrics

# Model evaluation included the area under the ROC curve (AUC), Brier score, and log-loss. The XGB model achieved:

# AUC = 0.916 (training)

# AUC = 0.844 (validation)

# 4. Feature Importance Assessment

# Importance was ranked using gain, cover, and frequency. NT-ProBNP was identified as the top predictor (gain = 0.216).

# 5. Analytical Considerations

# The Boruta algorithm may underrepresent features with weak associations in imbalanced datasets (e.g., 14% mortality rate). Additionally, although early stopping was implemented, the model remains susceptible to overfitting due to limited event numbers.

# Supplementary Tables

**Supplementary Table 1.** Comparison of baseline characteristics between the training and validation sets.

| Variables | Training set (n=441) | Validation set (n=189) | p |
| --- | --- | --- | --- |
| death,n(%) |  |  | 0.414 |
| NO | 374(84.8) | 165(87.3) |  |
| YES | 67(15.2) | 24(12.7) |  |
| Sex, % |  |  | 0.662 |
| female | 197(44.7) | 88(46.6) |  |
| male | 244(55.3) | 101(53.4) |  |
| Ethnic group, % |  |  | 0.458 |
| Han Chinese | 163(37) | 64(33.9) |  |
| Ethnic minorities | 278(63) | 125(66.1) |  |
| Age (years) | 0.6(0.3,1.7) | 0.7(0.3,1.8) | 0.165 |
| NYHA, % |  |  | 0.849 |
| 1 | 61(13.8) | 26(13.8) |  |
| 2 | 196(44.4) | 80(42.3) |  |
| 3 | 45(10.2) | 17(9) |  |
| 4 | 139(31.5) | 66(34.9) |  |
| Dyspnea, % |  |  | 0.398 |
| NO | 336(76.2) | 138(73) |  |
| YES | 105(23.8) | 51(27) |  |
| CHD, % |  |  | 0.325 |
| NO | 387(87.8) | 171(90.5) |  |
| YES | 54(12.2) | 18(9.5) |  |
| consciousness, % |  |  | 1.000 |
| NO | 378(85.7) | 162(85.7) |  |
| YES | 63(14.3) | 27(14.3) |  |
| LE-Edema, % |  |  | 0.487 |
| NO | 406(92.1) | 177(93.7) |  |
| YES | 35(7.9) | 12(6.3) |  |
| Card-Murmur, % |  |  | 0.690 |
| NO | 285(64.6) | 119(63) |  |
| YES | 156(35.4) | 70(37) |  |
| Lung-Moist, % |  |  | 0.244 |
| NO | 211(47.8) | 100(52.9) |  |
| YES | 230(52.2) | 89(47.1) |  |
| HR (bpm) | 145.6±29.9 | 143.7±26.6 | 0.434 |
| Fever, % |  |  | 0.618 |
| NO | 292(66.2) | 129(68.3) |  |
| YES | 149(33.8) | 60(31.7) |  |
| RR (breaths/min) | 40.3±14.7 | 38.8±13.0 | 0.231 |
| DBP (mmHg) | 55.5±13.2 | 56.0±13.2 | 0.665 |
| SBP (mmHg) | 91.3±15.9 | 92.3±15.1 | 0.473 |
| BMI (kg/m²) | 15.3±2.7 | 15.9±2.6 | 0.017 |
| WBC (×10⁹/L) | 11.4(8.1,15.7) | 11.5(7.7,15.9) | 0.874 |
| RBC (×10¹²/L) | 4.0±0.9 | 4.1±1.0 | 0.655 |
| Lymph (×10⁹/L) | 4.6(2.9,6.6) | 4.4(2.6,7.0) | 0.608 |
| Mono (×10⁹/L) | 0.8(0.5,1.2) | 0.9(0.6,1.3) | 0.712 |
| Neut (×10⁹/L) | 4.4(2.5,7.4) | 4.5(2.3,7.7) | 0.889 |
| Hb (g/L) | 102.0±21.8 | 102.7±25.2 | 0.728 |
| PLT (×10⁹/L) | 335.1±162.5 | 345.8±180.4 | 0.465 |
| ALT (U/L) | 27.0(18.0,52.9) | 25.1(16.0,38.0) | 0.057 |
| AST (U/L) | 47.3(34.9,78.5) | 47.3(34.4,70.0) | 0.371 |
| GGT (U/L) | 27.5(14.1,52.0) | 22.3(13.0,45.8) | 0.252 |
| DBIL (μmol/L) | 1.8(0.3,3.6) | 1.6(0.3,3.3) | 0.354 |
| IBIL (μmol/L) | 7.1(4.4,11.4) | 6.4(3.6,10.9) | 0.067 |
| ALB (g/L) | 36.3±6.9 | 36.4±7.6 | 0.848 |
| GLO (g/L) | 24.3±6.3 | 24.8±7.0 | 0.429 |
| Crea (μmol/L) | 26.0(19.4,34.0) | 25.0(19.8,34.0) | 0.911 |
| UA (μmol/L) | 292.2±163.1 | 258.4±143.7 | 0.014 |
| TC (mmol/L) | 3.1±1.4 | 3.1±1.1 | 0.870 |
| TG (mmol/L) | 1.3(0.9,1.7) | 1.3(1.0,1.7) | 0.611 |
| HDL-C (mmol/L) | 0.8±0.4 | 0.9±0.4 | 0.280 |
| LDL-C (mmol/L) | 1.9±1.1 | 1.8±0.8 | 0.443 |
| K^+^ (mmol/L) | 4.1±0.7 | 4.2±0.9 | 0.369 |
| Na^+^ (mmol/L) | 136.2±6.1 | 136.5±5.5 | 0.516 |
| Cl^-^ (mmol/L) | 102.5±7.3 | 102.8±6.5 | 0.635 |
| Ca (mmol/L) | 2.3±0.3 | 2.3±0.2 | 0.709 |
| P (mmol/L) | 1.6±0.6 | 1.6±0.6 | 0.853 |
| Mg^2+^ (mmol/L) | 0.9±0.2 | 0.9±0.2 | 0.138 |
| Glu (mmol/L) | 5.7±2.8 | 5.2±1.8 | 0.042 |
| LDH (U/L) | 363.2(273.0,562.9) | 347.0(273.8,519.9) | 0.370 |
| ALP (U/L) | 174.3(124.9,248.0) | 170.0(119.8,246.4) | 0.727 |
| ChE (U/L) | 5388.9±2052.5 | 5649.3±2370.4 | 0.165 |
| Cys-C (μmol/L) | 1.1±0.5 | 1.2±0.6 | 0.158 |
| TT (s) | 25.3±6.7 | 24.5±4.1 | 0.112 |
| PT (s) | 14.7±9.6 | 14.0±6.2 | 0.330 |
| D-Dimer (μg/L) | 676.0(313.0,1927.0) | 645.0(270.0,1913.0) | 0.439 |
| pH | 7.4±0.2 | 7.4±0.2 | 0.501 |
| GSP (mmol/L) | 2.1±0.7 | 2.1±0.5 | 0.935 |
| PCT (ng/mL) | 0.2(0.1,1.6) | 0.2(0.1,2.1) | 0.502 |
| NT-ProBNP (pg/mL) | 2866.0(1520.0,5089.0) | 2743.0(1397.0,4538.0) | 0.353 |
| CK (U/L) | 92.1(47.6,231.0) | 81.0(45.5,161.1) | 0.267 |
| Ccr (mL/min) | 49.4(30.5,75.0) | 50.5(35.1,72.3) | 0.570 |
| GFR (mL/min/1.73m²) | 754.7(455.8,1065.1) | 682.0(456.5,1003.7) | 0.467 |
| SV (mL) | 15.9±10.4 | 17.6±10.7 | 0.055 |
| CO (L/min) | 1.9±1.2 | 2.1±1.4 | 0.074 |
| LAD (mm) | 17.3±5.4 | 17.9±5.5 | 0.238 |
| LVESD (mm) | 17.3±6.9 | 18.1±7.5 | 0.228 |
| LVEDD (mm) | 25.5±7.9 | 26.7±8.4 | 0.076 |
| LVEF (%) | 63.9±12.2 | 63.4±12.1 | 0.629 |
| IVS Thickness (mm) | 4.3±1.4 | 4.3±1.1 | 0.964 |
| LVPW Thickness (mm) | 4.2±1.2 | 4.3±1.2 | 0.502 |
| RAD (mm) | 19.2±6.0 | 19.3±5.7 | 0.842 |
| RV Internal Dim (mm) | 11.4±3.4 | 11.2±3.2 | 0.652 |

**Supplementary Table 2.** Baseline characteristics of children under 5 years with heart failure(external validation dataset)

| Variables | Survival Group (n = 62) | Death Group (n = 11) | *P* |
| --- | --- | --- | --- |
|  |  |  |  |
| WBC(×10⁹/L) | 8.30 (6.68, 12.93) | 14.57 (7.67, 22.15) | 0.185 |
| PLT (×10⁹/L) | 285.00 (187.50, 373.00) | 135.00 (75.50, 330.50) | 0.257 |
| LDH(U/L) | 274.87 (231.16, 335.67) | 318.00 (177.44, 570.45) | 0.817 |
| pH | 6.00 (5.50, 6.50) | 6.00 (5.50, 6.00) | 0.309 |
| PCT(ng/mL) | 0.16 (0.07, 0.78) | 1.28 (0.25, 7.84) | 0.006 |
| NT ProBNP (pg/mL) | 2880.00 (448.42, 7061.50) | 558.00 (380.50, 605.00) | 0.013 |
| Crea(μmol/L) | 34.03 (28.69, 40.65) | 29.00 (27.23, 49.60) | 0.711 |

# Supplementary Figures

#
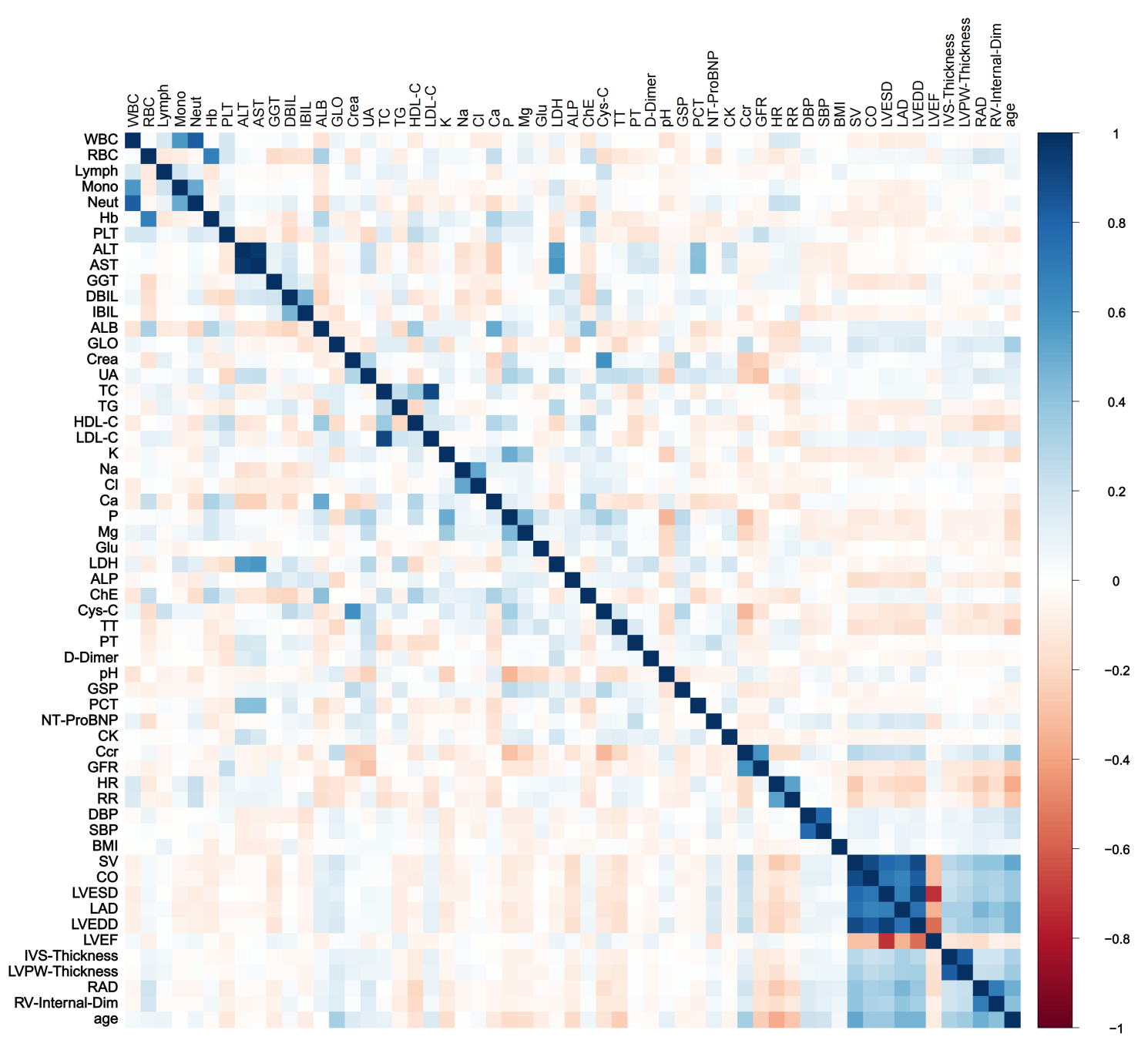


**Supplementary Figure 1.** Spearman correlation plot of 57 features. The color spectrum, ranging from blue to red represents the degree of correlation: closer to blue indicates a stronger positive correlation, while closer to red indicates a stronger negative correlation


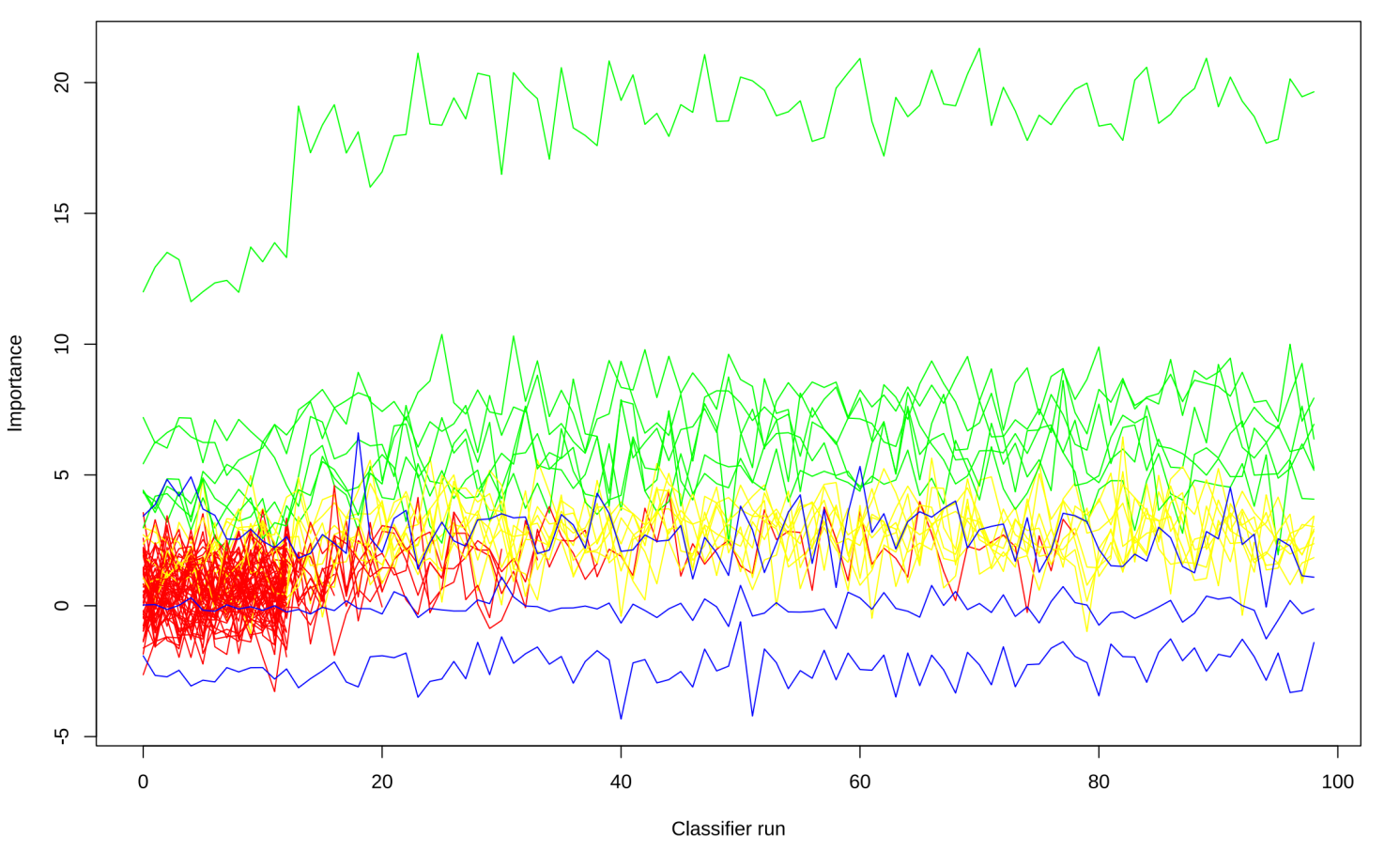


**Supplementary Figure 2.** Feature Importance Changes Across Multiple Classifier Runs in Boruta Model


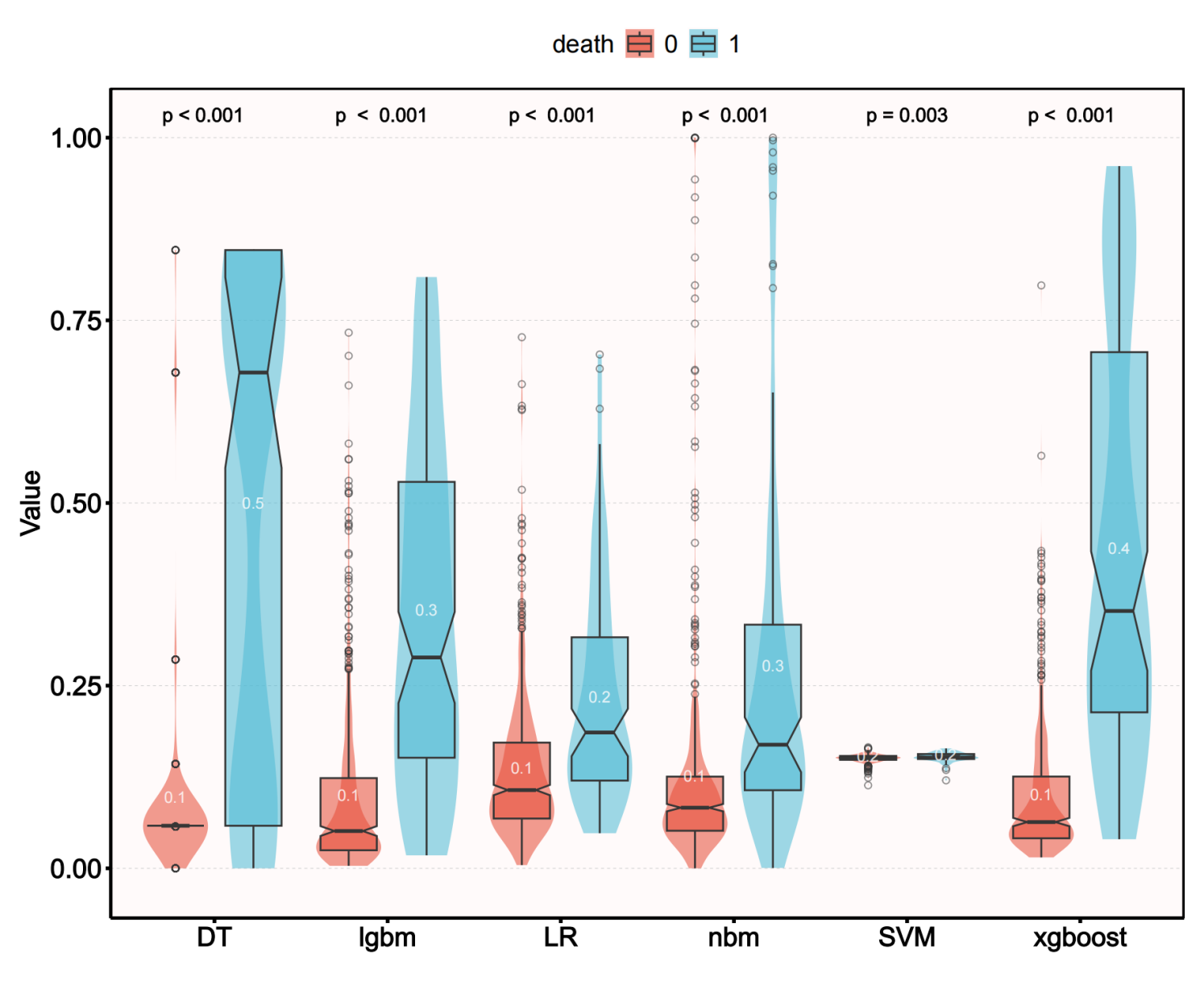


**Supplementary Figure 3.** Comparison of feature importance across different machine learning models.The x-axis represents the different models, while the y-axis shows the feature importance values. Each model's feature importance is visualized as a violin plot, with the median value indicated by a horizontal line within the box.
